# Dual Pathways of UBE4B Inhibit Apoptosis in p53-Positive Tumor Cells via CCAR2 Degradation

**DOI:** 10.3390/ijms27083682

**Published:** 2026-04-21

**Authors:** Bo Jin, Junyao Qu, Peng Xu, Bo Zhao, Xianting Jiao

**Affiliations:** 1 Department of Pediatric Infectious, Xinhua Hospital Affiliated to Shanghai Jiao Tong University School of Medicine, Shanghai 200092, China; 2 Engineering Research Center of Cell and Therapeutic Antibody, Ministry of Education, School of Pharmacy, Shanghai Jiao Tong University, Shanghai 200240, China

**Keywords:** ubiquitin, ubiquitination, degradation, UBE4B, p53, CCAR2, cell apoptosis, dual regulatory

## Abstract

Apoptosis, or programmed cell death, is a fundamental process essential for tissue homeostasis, development, and the elimination of damaged or potentially cancerous cells. Here, we identify the E3/E4 ubiquitin ligase UBE4B as a critical suppressor of apoptosis in p53-proficient tumor cells, functioning through a previously uncharacterized dual mechanism. Initially, an orthogonal ubiquitin transfer screening approach identified CCAR2 as a UBE4B substrate. We demonstrate that UBE4B interacts with and ubiquitinates CCAR2, promoting its proteasomal degradation. Furthermore, we found that UBE4B concurrently targets p53 itself for ubiquitin-dependent degradation. Functionally, UBE4B overexpression suppresses apoptosis, whereas rescue experiments indicate that restoring p53 expression reverses this suppression more effectively than restoring CCAR2, highlighting the dominance of the direct p53 degradation pathway. Mechanistically, UBE4B deficiency leads to CCAR2 accumulation, which inhibits SIRT1 activity, thereby enhancing p53 acetylation and stability; this effect is reversed upon CCAR2 co-depletion. Consistently, transcriptional profiling confirms that UBE4B downregulates key p53 target genes (e.g., BAX, PUMA) through this dual-pathway regulation. In summary, our study establishes that UBE4B acts as a key apoptosis suppressor by coordinately degrading both p53 and its positive regulator CCAR2, revealing a targetable vulnerability in p53-wild-type tumors.

## 1. Introduction

Apoptosis, also known as programmed cell death, is an essential biological process for development and homeostasis in multicellular organisms [1,2,3]. Its impairment is intimately linked to various diseases, such as cancer, autoimmunity and neurodegeneration [4]. The process of apoptosis is primarily mediated by two pathways: the extrinsic pathway [5], initiated by the binding of death ligands like TNF-α [6,7] and TRAIL [8,9,10] to their specific cell-surface receptors, and the intrinsic pathway [11,12], which is activated in response to intracellular stress signals like DNA damage [13], oxidative stress [14] and oncogene activation [15].

The tumor suppressor p53 functions as a master transcriptional regulator of apoptosis. It orchestrates cell death by activating a broad network of pro-apoptotic target genes involved in both the intrinsic and extrinsic pathways [16,17,18]. Upon potent activation by DNA damage, p53 transcriptionally upregulates key pro-apoptotic genes [10,19,20], thereby initiating the apoptotic program. Although wild-type p53 remains intact in many cancers [21], its pro-apoptotic function is frequently suppressed by upstream regulators.

Cell cycle and apoptosis regulator 2(CCAR2/DBC1) is a multifunctional nuclear protein that regulates diverse cellular processes. It functions as a co-activator for several transcription factors (e.g., p53 [22], ERα [23], AR [24]) and as a direct inhibitor of multiple epigenetic and regulatory enzymes, including the deacetylases SIRT1 [25] and HDAC3 [26].

Within the DNA damage response, a core function of CCAR2 is to modulate p53 activity by binding to and inhibiting SIRT1, an NAD^+^-dependent deacetylase [27,28]. Under DNA damage conditions, CCAR2 binds to the catalytic domain of SIRT1, blocking SIRT1-mediated deacetylation of p53. This leads to increased acetylation of p53, thereby enhancing its transcriptional activity and activating the expression of pro-apoptotic genes, ultimately inducing apoptosis in tumor cells [25,29]. This mechanism establishes the tumor-suppressive role of CCAR2 in the SIRT1-p53 pathway.

As a crucial post-translational modification, ubiquitination is an enzymatic cascade involving E1, E2, and E3 ligases that plays a central role in vital cellular processes such as apoptosis by precisely regulating the stability of key proteins [30,31]. Ubiquitin signaling not only directs substrates for degradation via the proteasome or lysosome but also functions as a versatile signaling mechanism, extensively regulating diverse cellular processes including the cell cycle and DNA damage response [32]. However, due to the vast number of E3 ubiquitin ligases and the high specificity of their substrate recognition mechanisms, identifying the direct physiological substrates of individual E3 enzymes remains a major challenge in the field [33,34]. To address this, our team previously developed the orthogonal ubiquitin transfer (OUT) technology, which enables the systematic and high-confidence identification of direct substrates for specific E3 ubiquitin ligases [35,36]. Using this platform, we have successfully identified a series of potential substrates for several E3 enzymes, including CHIP [37,38], UBE4B [37,39], E6AP [40,41,42] and Rsp5 [43,44]. Among these, UBE4B has been shown to regulate multiple cellular processes such as DNA damage response [45]. Notably, several key proteins, including p53 [46], Ataxin-3 [47] and FAT4 [48], have been established as canonical substrates of UBE4B. In our prior screen of HEK293T cells using the OUT technology, CCAR2 was identified as one of over 100 candidate substrates of UBE4B [37]. This finding identifies CCAR2 as a potential novel downstream target of UBE4B, suggesting its involvement in the apoptotic regulatory network via a new pathway.

Our preliminary observations (unpublished data) revealed that overexpression of UBE4B inhibits apoptosis in H125 cells (p53-wild-type tumor cells) but not in H1299 cells (p53-null cells), indicating that its apoptotic-inhibiting effect depends on functional p53. However, the underlying mechanism remained unclear. Based on this background, the present study aimed not only to validate CCAR2 as a novel substrate of UBE4B, but also to define a comprehensive regulatory model. Specifically, we demonstrate that UBE4B employs a novel two-pronged mechanism to suppress apoptosis: first, through its canonical function of mediating p53 degradation [46,49,50]; second, by targeting CCAR2 for degradation, which relieves CCAR2-mediated inhibition of SIRT1. Consequently, SIRT1 deacetylase activity is enhanced, leading to reduced acetylation and transcriptional activity of p53, and ultimately to indirect suppression of the apoptotic program. This dual-targeting regulatory model integrates UBE4B, CCAR2, SIRT1, and p53 into a coherent signaling network for the first time. Our findings establish CCAR2 as a key downstream target of UBE4B and elucidate the dual-pathway mechanism by which UBE4B orchestrates apoptotic resistance in p53-positive tumors, providing a new conceptual framework for therapeutic intervention.

## 2. Results

### 2.1. UBE4B Interacts with CCAR2

**Figure 1:**
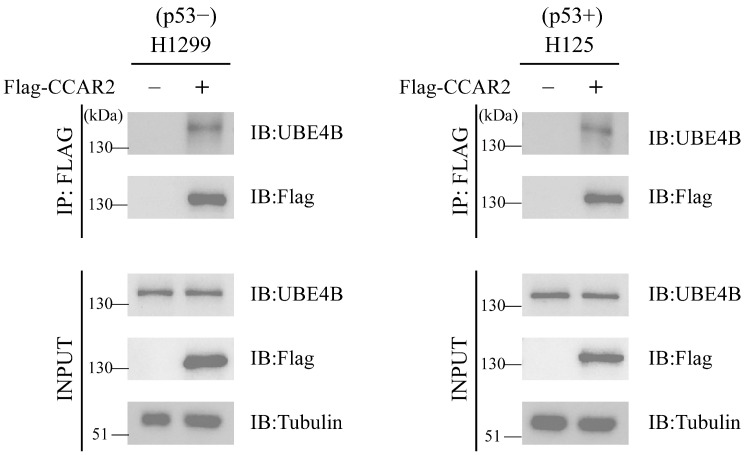
UBE4B interacts with CCAR2. H1299 and H125 cells were transfected with Flag-CCAR2 (2 μg) or an empty vector (2 μg) plasmid as a control for 48 h. The interaction between UBE4B and CCAR2 was assessed by co-immunoprecipitation (Co-IP). Specifically, the assay was performed using an anti-FLAG antibody and an anti-UBE4B antibody, respectively. Tubulin levels served as the internal reference.

Based on our previous identification of CCAR2 as a potential substrate of UBE4B [37], we further validated their direct interaction through co-immunoprecipitation (Co-IP) assays in H125 (p53-proficient) and H1299 (p53-null) tumor cell lines. Both cell lines were transfected with FLAG-tagged CCAR2 expression plasmids, while control groups received empty vector plasmid. Following immunoprecipitation with anti-FLAG antibodies, endogenous UBE4B was detected in FLAG-CCAR2-transfected groups from both cell lines. No bands were observed in empty vector groups (Figure 1). These results confirm a specific physical interaction between UBE4B and CCAR2.

### 2.2. UBE4B Promotes the Ubiquitination of CCAR2

**Figure 2:**
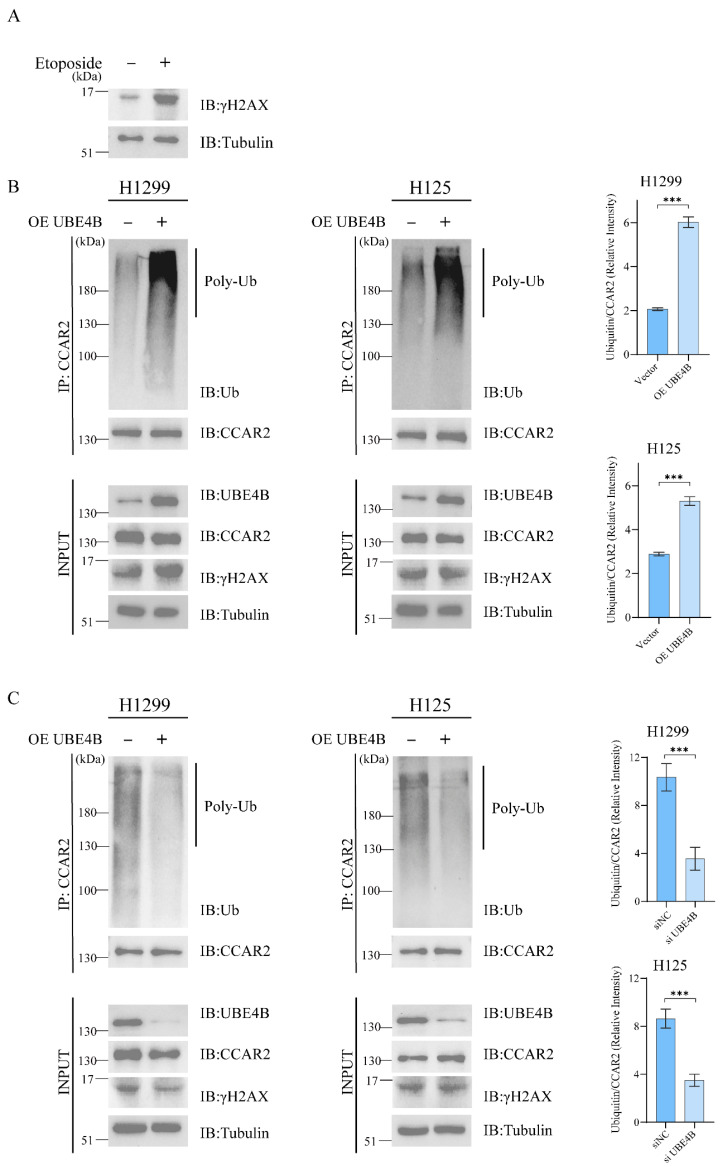
UBE4B promotes the ubiquitination of CCAR2. (**A**) To induce DNA damage, cells were treated with etoposide (5 μM) for 4 h. This treatment condition was applied to all subsequent cellular experiments. The treated cells were then subjected to immunoblot analysis. (**B**,**C**) H1299 and H125 cells were transfected with either a UBE4B overexpression plasmid (2 μg) or an empty vector control (2 μg) for 48 h (**B**) and transfected with either siUBE4B or siNC for 60 h (**C**) followed by a 4 h treatment with MG132 (10 μM). The ubiquitination of CCAR2 was detected by Co-IP with the anti-CCAR2 and anti-Ub antibodies. Data are shown as mean ± SD (*n* = 3). Statistical significance: *** *p* < 0.001.

To determine if UBE4B regulates ubiquitination of its binding partner CCAR2, we performed Co-IP assays. We conducted all subsequent experiments in etoposide-treated cells to induce DNA damage. Increased γ-H2AX intensity, a canonical DNA damage marker [51], confirmed successful establishment of the DNA damage model (Figure 2A).

We transfected two distinct cell lines with empty vector or UBE4B-expressing plasmid, followed by immunoprecipitation of endogenous CCAR2. Western blotting with anti-ubiquitin antibodies revealed polyubiquitinated CCAR2 species, which were markedly enhanced upon UBE4B overexpression compared to controls (Figure 2B). Consistent results across both cell lines demonstrate that UBE4B promotes CCAR2 ubiquitination.

### 2.3. UBE4B Mediates the Degradation of CCAR2

**Figure 3:**
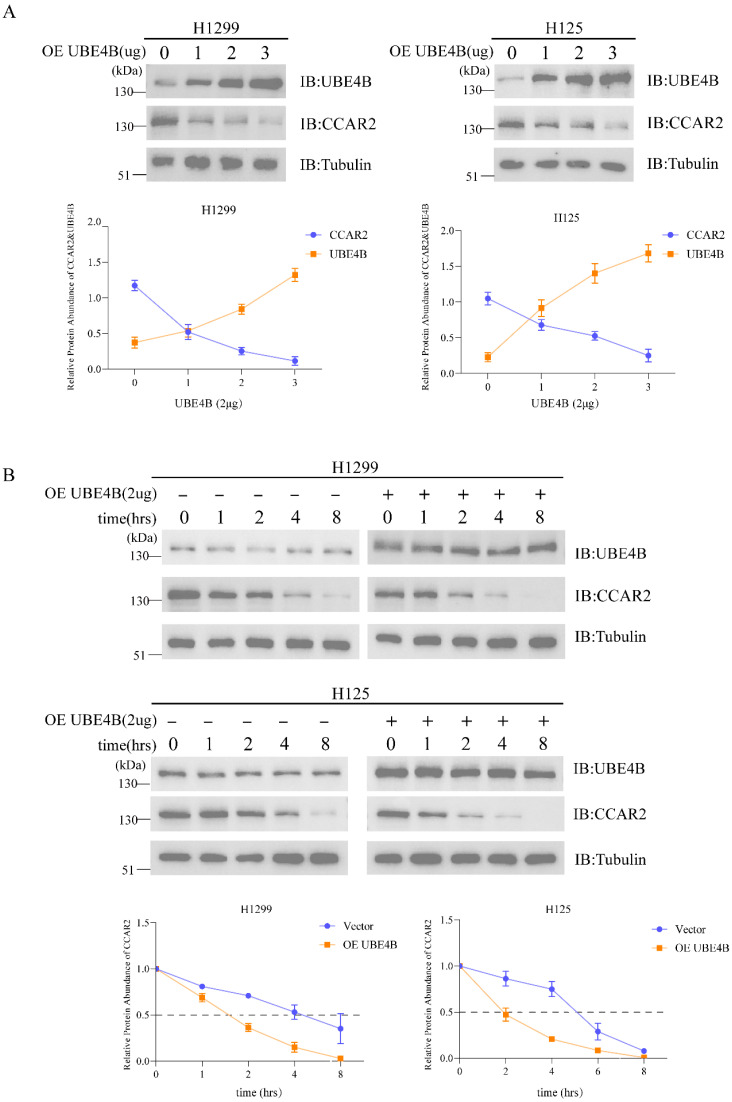
UBE4B mediates the degradation of CCAR2. (**A**) After treatment with etoposide (5 μM), H1299 and H125 cells were transfected with different amounts of UBE4B for 48 h. CCAR2 degradation caused by UBE4B is detected by Western blotting with the anti-UBE4B and anti-CCAR2 antibodies. (**B**) After treatment with etoposide (5 μM), H1299 and H125 cells were transfected with either a UBE4B overexpression plasmid (2 μg) or an empty vector control (2 μg) for 48 h, and then treated with CHX (10 μM) for 0, 1, 2, 4, 8 h. The degradation of CCAR2 caused by UBE4B is detected by Western blotting with the anti-UBE4B and anti-CCAR2 antibodies. The half-life of CCAR2 was indicated by dotted lines.

To investigate the regulatory role of UBE4B on CCAR2 protein stability, we performed dose-dependent transfection experiments in two cell lines under DNA damage conditions. Cells were transfected with increasing concentrations of UBE4B-expressing plasmid (0, 1, 2, and 3 μg). Western blot analysis revealed a dose-dependent reduction in endogenous CCAR2 protein levels upon UBE4B overexpression (Figure 3A), indicating that UBE4B promotes CCAR2 degradation through ubiquitination-mediated proteolysis.

To further evaluate the impact of UBE4B on CCAR2 stability, we measured CCAR2 half-life using cycloheximide (CHX) chase assays. Following protein synthesis inhibition, CCAR2 levels were monitored over time. In control cells, CCAR2 exhibited a half-life of approximately 4 h. In contrast, UBE4B-overexpressing cells showed accelerated degradation kinetics, with the half-life shortened to 2 h (Figure 3B). This effect was consistently observed in both cell lines, further validating UBE4B-mediated regulation of CCAR2 turnover.

### 2.4. UBE4B Regulates Apoptosis Through Its Effects on CCAR2 and p53 in p53-Positive Cells

**Figure 4:**
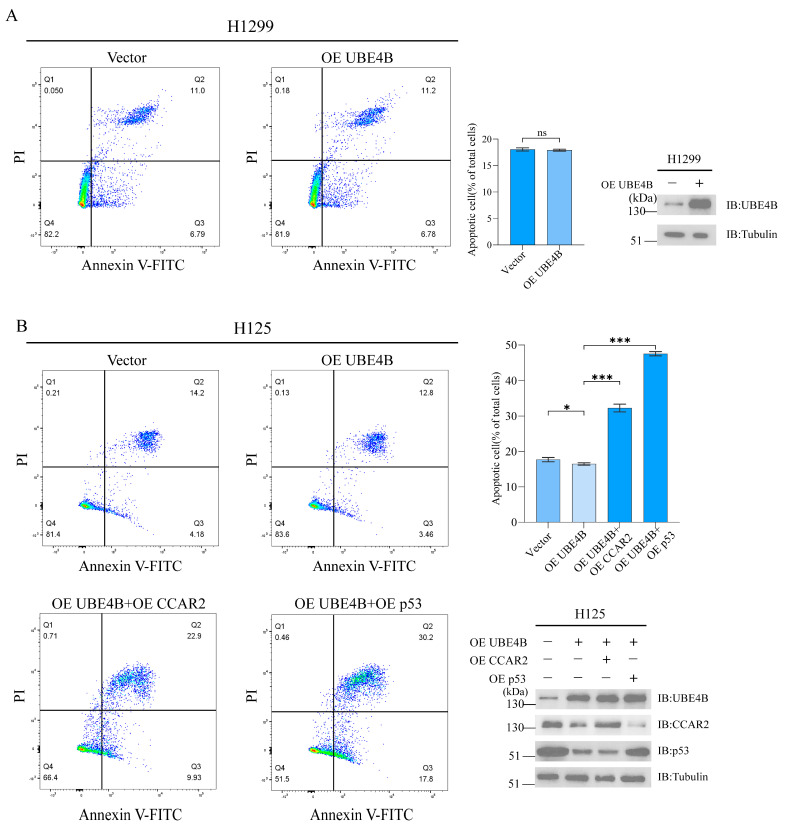
UBE4B regulates apoptosis through its effects on CCAR2 and p53 in p53-positive cells. (**A**,**B**) After treatment with etoposide (5 μM), H1299 and H125 cells were transfected with UBE4B (2 μg) alone or together with CCAR2 (2 μg) or p53 (2 μg) and cultured with NAD^+^ (50 μM). The populations of early apoptotic (Annexin V-FITC+/PI−, Q3) and late apoptotic (Annexin V-FITC+/PI+, Q2) cells were then quantified. The expression level of the transfected plasmid was assessed by immunoblotting (Western blot) analysis. Data are shown as mean ± SD (*n* = 3). Statistical significance: * *p* < 0.05; *** *p* < 0.001; “ns” indicates a non-significant result.

To clarify the role of the UBE4B-CCAR2-p53 axis in apoptosis regulation, we overexpressed UBE4B in DNA-damaged H1299 (p53-null) and H125 (p53-proficient) cells. Flow cytometry (Annexin V-FITC/PI staining) revealed that UBE4B overexpression had no significant effect on apoptosis in H1299 cells (apoptotic rate: 17.79% control vs. 17.98% OE UBE4B) (Figure 4A), while it inhibited apoptosis in H125 cells (apoptotic rate decreased from 18.38% to 16.26%), confirming that UBE4B-mediated apoptosis inhibition strictly depends on the p53 pathway (Figure 4B).

To further explore the mechanism of UBE4B-induced apoptosis inhibition in p53-proficient cells, rescue experiments in H125 cells showed that co-expression of UBE4B and CCAR2 increased the apoptosis rate to 32.83%, while co-transfection of UBE4B and p53 further elevated apoptosis to 48.0%. Western blotting confirmed successful overexpression of UBE4B, CCAR2, and p53 in both cell lines (Figure 4A,B).

Notably, p53 rescue exhibited stronger pro-apoptotic effects than CCAR2, indicating that direct degradation of p53 dominates UBE4B-mediated apoptosis suppression. Overexpression of p53 more effectively restored pro-apoptotic transcriptional function, while CCAR2 overexpression only rescued the acetylation level of non-degraded p53 due to concurrent UBE4B-mediated degradation of p53, limiting the efficiency of this pathway. Combined analysis reveals that UBE4B employs a dual mechanism to suppress apoptosis in p53-proficient tumor cells: on the one hand, it directly mediates ubiquitination-dependent degradation of p53 protein; on the other hand, it degrades CCAR2 to relieve its inhibition of SIRT1, thereby enhancing SIRT1 deacetylase activity and reducing p53 acetylation.

### 2.5. UBE4B Regulates p53 Acetylation Through the CCAR2-SIRT1 Pathway

**Figure 5:**
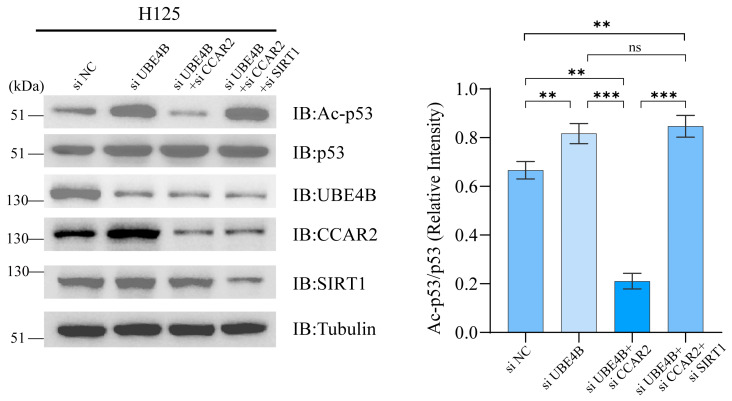
UBE4B regulates p53 acetylation through the CCAR2-SIRT1 pathway. After treatment with etoposide (5 μM), H125 cells were transfected with siRNAs for 60 h and cultured with NAD+ (50 μM). Acetylated p53 levels were assessed by Western blot analysis using specific antibodies, and the band intensity was normalized to that of total p53. Data are shown as mean ± SD (*n* = 3). Statistical significance: ** *p* < 0.01; *** *p* < 0.001; “ns” indicates a non-significant result.

To clarify the mechanism by which UBE4B regulates the SIRT1-p53 acetylation axis through CCAR2 degradation, we conducted further experiments in p53-wild-type H125 cells. Since UBE4B overexpression degrades both CCAR2 and p53 protein, interfering with acetylation detection, this study employed siUBE4B to specifically block its expression and established four groups: siNC control group, siUBE4B group, siUBE4B + s iCCAR2 co-transfection group, and siUBE4B + siCCAR2 + siSIRT1 co-transfection group. Western blot analysis showed that CCAR2 protein accumulated in the siUBE4B group, while total p53 protein increased due to reduced degradation. Detection with an acetylated p53 antibody (Lys382) revealed that p53 acetylation (K382 site) significantly increased after UBE4B knockdown; however, in the siUBE4B + siCCAR2 co-transfection group, p53 acetylation levels markedly decreased. To determine whether the reduction in p53 acetylation resulted from restored SIRT1 deacetylase activity following CCAR2 degradation, we performed additional knockdown of SIRT1 and found that p53 acetylation was restored, indicating that SIRT1 mediates the deacetylation following CCAR2 degradation (Figure 5). Collectively, the enhanced p53 acetylation following UBE4B knockdown, its reversal upon CCAR2 co-depletion, and subsequent rescue by SIRT1 inhibition together demonstrate that UBE4B modulates the SIRT1-p53 axis via CCAR2 degradation to regulate apoptosis.

### 2.6. UBE4B Reduces p53-Dependent Transcription of Pro-Apoptotic Genes by Degrading CCAR2

**Figure 6:**
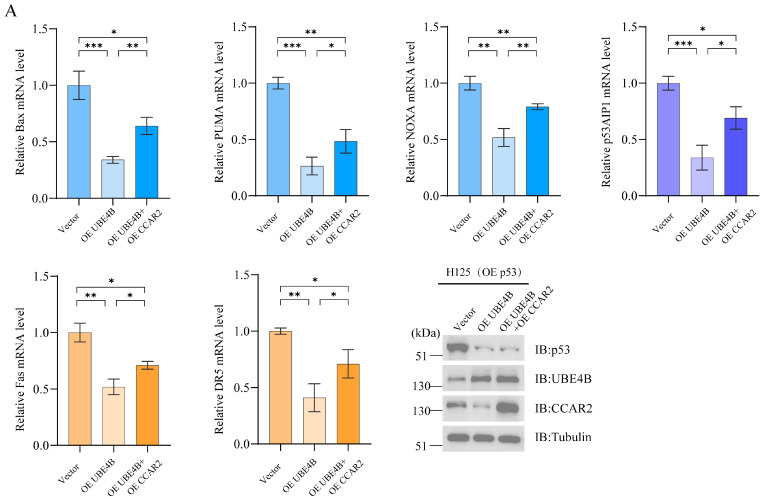

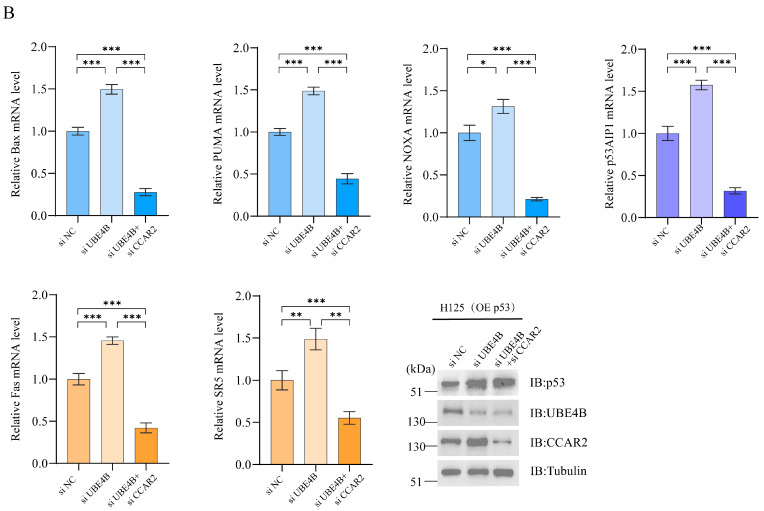
UBE4B reduces p53-dependent transcription of pro-apoptotic genes by degrading CCAR2. (**A**,**B**) After treatment with etoposide (5 μM), H125 cells were transfected with either siRNAs for 60 h with NAD+ (50 μM). The mRNA levels of p53-regulated downstream genes were quantified by qPCR. The expression level of the transfected siRNA was assessed by immunoblotting analysis. Data are shown as mean ± SD (*n* = 3). Statistical significance: * *p* < 0.05; ** *p* < 0.01; *** *p* < 0.001.

Alterations in p53 acetylation levels lead to corresponding changes in the transcriptional activity of its downstream genes [25]. Here, we performed qPCR analysis for several canonical p53 target genes: the pro-apoptotic Bcl-2 family members Bax, PUMA, and NOXA; the death receptors Fas and DR5; and other apoptosis-associated genes including p53AIP1. To ensure sufficient p53 levels in H125 cells for influencing downstream apoptotic genes, we transfected exogenous p53 into DNA-damaged H125 cells and established experimental groups for validation. All data were normalized. Results showed that transcriptional levels of apoptosis-related genes across different pathways decreased in the OE UBE4B group, partially recovered in co-transfection groups but remained lower than controls (Figure 6A). This occurred because OE CCAR2 restored p53 acetylation but failed to restore p53 protein levels degraded by OE UBE4B. In siRNA groups, transcriptional levels of apoptotic genes increased in the siUBE4B group but significantly decreased after siCCAR2 co-transfection (Figure 6B). These results collectively demonstrate that UBE4B affects p53-regulated transcription of apoptotic genes through dual pathways: directly degrading p53 and indirectly influencing CCAR2.

## 3. Discussion

This study demonstrates for the first time that UBE4B inhibits apoptosis in p53-proficient tumor cells via a dual molecular mechanism. In the previously reported direct pathway, functioning as an E3/E4 ubiquitin ligase, UBE4B binds to the C-terminal domain of p53 through its conserved U-box domain and cooperates with Hdm2 to catalyze polyubiquitination of p53 [46]. This process markedly shortens p53 half-life, thereby directly blocking its transcriptional activation function, such as inhibiting the expression of pro-apoptotic genes like BAX and PUMA. Beyond this established mechanism, the key novelty of our study lies in the identification of an indirect pathway: UBE4B mediates ubiquitin-dependent degradation of CCAR2, which relieves CCAR2-mediated inhibition of SIRT1. Consequently, the enhanced deacetylase activity of SIRT1 reduces p53 acetylation [25]. The loss of this acetylation modification impairs the ability of p53 to bind DNA response elements, ultimately suppressing the transcription of pro-apoptotic genes. To our knowledge, this is the first report identifying CCAR2 as a direct substrate of UBE4B and establishing the UBE4B-CCAR2-SIRT1-p53 acetylation axis. This dual regulatory model reveals a hierarchical integration of the ubiquitination system and epigenetic modification within the p53 pathway—where UBE4B achieves coordinated suppression of apoptosis by synchronously degrading p53 and CCAR2. Functional validation showed that UBE4B overexpression reduced apoptosis in H125 cells, and this effect was fully reversed by CCAR2 or p53 co-expression. However, the observed anti-apoptotic effect was modest, with the apoptotic rate only decreasing from 18.38% to 16.26% (*p* < 0.05). This modest reduction is likely attributable to a ceiling effect caused by high-dose etoposide treatment, which elevated baseline apoptosis to approximately 20% and left limited dynamic range for further modulation. Importantly, the complete reversal of this effect by CCAR2 or p53 co-expression supports the functional specificity of the UBE4B-CCAR2-p53 axis despite the modest magnitude.

Notably, the identification of CCAR2 as a novel substrate of UBE4B holds significant molecular biological implications. Through orthogonal ubiquitin transfer (OUT) screening, CCAR2 was identified as a direct substrate of UBE4B [37]. Unlike other known E3 ligases, UBE4B is the first E3/E4 ubiquitin ligase confirmed to simultaneously target both p53 and CCAR2, a discovery that fills a mechanistic gap in understanding CCAR2 stability regulation. Meanwhile, the function of CCAR2 exhibits p53 status dependence. In p53-wild-type tumors, CCAR2 acts as a tumor suppressor by inhibiting SIRT1, and its degradation leads to insufficient p53 acetylation; whereas in p53-mutant tumors, CCAR2 stabilizes mutant p53 and enhances its oncogenic activity, such as activating the Wnt pathway [52]. In this study, the weaker ability of CCAR2 rescue to restore apoptosis compared to p53 rescue is likely attributable to reasons such as the real-time degradation of newly synthesized CCAR2 by UBE4B and a reduction in total p53 protein levels. This observation confirms the tumor-suppressive role of CCAR2 in the p53-wild-type context, while also hinting at the limiting impact of persistent UBE4B-mediated p53 degradation.

While this study establishes the UBE4B-CCAR2-p53 axis as a novel regulatory mechanism, several limitations should be acknowledged. First, mass spectrometry could be employed to precisely map the ubiquitination sites on CCAR2, and lysine-to-arginine mutants (K to R) could be constructed to validate their resistance to UBE4B-mediated degradation. Concurrently, the cooperative network between UBE4B and other E3 ligases requires further exploration. For instance, while the mechanism of UBE4B collaborating with MDM2 via RING domain interaction to degrade p53 is established, whether UBE4B antagonizes CHIP-mediated degradation of CCAR2 (where CHIP binds the Hsp70-CCAR2 complex via its TPR domain) still awaits experimental verification. Second, our conclusion that UBE4B regulates p53 acetylation via SIRT1 relies primarily on genetic co-knockdown experiments (siCCAR2 and siSIRT1). Direct biochemical evidence of SIRT1 deacetylase activity modulation—such as in vitro deacetylation assays using purified SIRT1 and acetylated p53 peptide in the presence of NAD^+^—is currently lacking. Future studies incorporating direct SIRT1 activity measurements will further substantiate the mechanistic model proposed here. Addressing these limitations will provide a more complete understanding of how UBE4B integrates ubiquitination and acetylation signaling to control apoptosis.

In summary, the discovery of the UBE4B-CCAR2-p53 axis presents a new paradigm for apoptosis regulation: UBE4B acts as a molecular hub, achieving dual blockade of the apoptotic pathway by synchronously degrading a transcription factor (p53) and its epigenetic regulator (CCAR2). This mechanism highlights the central role of the ubiquitination system in integrating protein stability and epigenetic modification, providing a theoretical framework for targeting E3/E4 ligases to restore p53 function.

## 4. Materials and Methods

### 4.1. Cell Culture and Reagents

Human lung cancer cell lines NCI-H125 and NCI-H1299 were used in this study. NCI-H125 cells (p53-positive) are from a stock maintained in our laboratory; the genetic information for this cell line is publicly available in the Cellosaurus database (accession number CVCL_3968). NCI-H1299 cells (p53-null) were obtained from the American Type Culture Collection (ATCC, Manassas, VA, USA, catalog No. CRL-5803™). They were maintained in Roswell Park Memorial Institute 1640 (RPMI 1640; Gibco, Waltham, MA, USA) supplemented with 10% fetal bovine serum (FBS; Gibco C11875500BT). They were cultured at 37 °C in a humidified environment with 5% CO2. The medium was replaced every 48 h.

Reagents: etoposide (MCE, Monmouth Junction, NJ, USA, HY-13629), CHX (MCE, HY-12320), NAD+ (MCE, HY-B0445).

### 4.2. Cell Transfection

H125 cells and H1299 cells were transfected with Lipofectamine™ 3000 (Invitrogen, Waltham, MA, USA, cat. no. L3000001) when they reached approximately 70% confluence. Plasmid transfection lasted for 48 h, while siRNA transfection was conducted for 24 h. The medium was replaced 24 h post-transfection.

### 4.3. siRNA and Antibodies

All the siRNA were ordered from Sangon Biotech. The siRNA sequences used in the manuscript were below:

siUBE4B: 5′-CUGCAAUGCUGAACUUUAATT-3′

siCCAR2: 5′-AAACGGAGCCUACUGAACAUU-3′

siSIRT1: 5′-ATGGAGAAACATGTTATATATAC-3′

The following antibodies were used for Western blotting: FLAG (Mouse monoclonal, Abcam, Cambridge, UK, ab49763, 1:1000), CCAR2 (Rabbit polyclonal, Proteintech, Rosemont, IL, USA, 22638-1-AP, 1:5000), UBE4B (Rabbit polyclonal, Abcam, ab97697, 1:5000), UB (Rabbit polyclonal, Abcam, ab134953, 1:5000), Tubulin (Mouse monoclonal, Proteintech, 66031-1-Ig, 1:5000), p53 (Rabbit monoclonal, Proteintech, 10442-1-AP, 1:5000), SIRT1 (Rabbit monoclonal, Proteintech, 13161-1-AP, 1:2000), γH2AX (Rabbit monoclonal, Proteintech, 83307-2-RR, 1:5000), Ac-p53 (Lys382) (Rabbit monoclonal, Abcam, ab75754, 1:1000)

### 4.4. Co-Immunoprecipitation (Co-IP)

Cells were lysed with RIPA I lysis buffer (Sangon Biotech, Shanghai, China, C500005-0100) supplemented with 100 × Protease Inhibitor Cocktail (MCE, HY-K0010), 100× Phenyl Methane Sulfonyl Fluoride (PMSF, Beyotime, Shanghai, China, ST507), and 100× Phosphatase Inhibitor Complex I (Sangon Biotech, C500017-0001). For immunoprecipitation, 1 mg of total protein was incubated with pre-cleared beads at 4 °C overnight. The beads were then washed three times with TBS-T (TBS, Sangon Biotech, B040126-0005; Tween-20, A631023-0500) buffer, and the bound proteins were eluted by boiling in SDS-PAGE loading buffer at 95 °C for 10 min. We used two kinds of beads in this expirement: Anti-Flag Tag Magnetic Beads (Sharebio, Shanghai, China, SB-PR002) and Protein A/G Magnetic Beads (Sharebio, SB-PR001).

### 4.5. Statistical Analysis

Protein band intensities from Western blots were quantified by measuring the grayscale value using ImageJ (1.54g). To control for loading variations, the grayscale value of each target protein band was normalized to that of its corresponding internal control band, yielding a relative intensity value. Data visualization was performed using GraphPad Prism 8 (10.1.2). For bar graphs, the calculated relative intensities were plotted directly. For the analysis in Figure 3B, the relative intensity of the CCAR2 at each treatment concentration was first normalized to its value at the 0 concentration point to determine the relative protein expression level, which was then presented in a line graph. Statistical significance was assessed using a two-tailed, unpaired Student’s t-test for comparisons between two groups. Data from three independent experimental replicates (*n* = 3) are presented as the mean ± standard deviation (SD). Significant differences are denoted as follows: * *p* < 0.05; ** *p* < 0.01; *** *p* < 0.001; “ns” indicates a non-significant result. Error bars on all graphs represent the ±SD.

### 4.6. Apoptosis Detection

Cell apoptosis was assessed by flow cytometry using an Annexin V-FITC/PI apoptosis detection kit (Biosharp, Beijing, China, BL107A). Cells were transfected with siRNAs for 72 h. After transfection, the cells were harvested by trypsinization (without EDTA) and washed twice with phosphate-buffered saline (PBS, Sangon Biotech, B640011-0010) via centrifugation at 800× g for 3 min per wash. For staining, approximately 5 × 105 cells were resuspended in 500 μL of PBS. The cell suspension was sequentially stained with 5 μL of Annexin V-FITC and 5 μL of propidium iodide (PI), with gentle mixing after each addition. The stained cells were incubated at room temperature in the dark for 10 min prior to analysis. Flow cytometric analysis was performed immediately after incubation. Annexin V-FITC fluorescence was detected using an excitation wavelength of 488 nm and an emission wavelength of 530 nm. PI fluorescence was detected with excitation at 488 nm and emission collected at wavelengths ≥ 630 nm.

### 4.7. RNA Extraction and Quantitative Real-Time PCR (qPCR)

Total RNA was extracted from H125 cells under the specified experimental conditions with TRIzol (Sangon Biotech, B610409-0100), following the manufacturer’s protocol. The concentration and purity of the isolated RNA were determined using a NanoDrop 2000 spectrophotometer (Thermo Scientific, Waltham, MA, USA). Samples with A260/A280 ratios ranging from 1.9 to 2.1 were used for subsequent analysis. Genomic DNA was removed, and first-strand cDNA was synthesized from 1 µg of total RNA using the PrimeScript RT reagent Kit with gDNA Eraser (Takara Bio, Kusatsu, Shiga, Japan, RR037A).

Quantitative PCR was carried out on a QuantStudio 6 Flex Real-Time PCR System (Applied Biosystems, Foster City, CA, USA) with TB Green Premix Ex Taq II (Takara Bio, RR420A). Each 20 µL qPCR reaction contained 10 µL of TB Green mix, 0.8 µL each of forward and reverse primers (10 μM), 2 µL of cDNA template, and 6.4 µL of nuclease-free water. The thermal cycling conditions were as follows: initial denaturation at 95 °C for 30 s, followed by 40 cycles of 95 °C for 5 s and 60 °C for 30 s. A melting curve analysis (60 °C to 95 °C) was performed after amplification to verify the specificity of the PCR products. The mRNA expression level of GAPDH was measured as an endogenous control for normalization. All reactions were run in technical triplicates. Relative gene expression was calculated using the comparative 2−ΔΔCt method. GraphPad Prism software (version 9.0) was used for statistical analysis. Differences between two groups were evaluated by an unpaired, two-tailed Student’s t-test. Data from three independent experimental replicates (*n* = 3) are presented as the mean ± standard deviation (SD). Significant differences are denoted as follows: * *p* < 0.05; ** *p* < 0.01; *** *p* < 0.001; “ns” indicates a non-significant result. Error bars on all graphs represent the ±SD.

The primers used in the manuscript were below:
BaxForward PrimerCCCGAGAGGTCTTTTTCCGAG
Reverse PrimerCCAGCCCATGATGGTTCTGATPUMAForward PrimerGACCTCAACGCACAGTACGAG
Reverse PrimerAGGAGTCCCATGATGAGATTGTNOXAForward PrimerCTCACCGTGTGTAGTTGGCA
Reverse PrimerACTTCCAGCTCCGCCGTAFasForward PrimerCGGAGTTGGGGAAGCTCTTT
Reverse PrimerTTTGGTGCAAGGGTCACAGTDR5Forward PrimerCTGCGCCCACAAAATACACC
Reverse PrimerTCCCCACTGTGCTTTGTACCp53AIP1Forward PrimerCTGGCTGGGTTTCAGATCCC
Reverse PrimerAAATGAGGTGGCCGCTAGTC

## Data Availability

The original contributions presented in this study are included in the article. Further inquiries can be directed to the corresponding authors.

## Abbreviations

CCAR2	Cell Cycle And Apoptosis Regulator
UBE4B	Ubiquitination Factor E4B
CHIP	C-terminus of Hsc70-Interacting Protein
E6AP	E6-Associated Protein
OUT	Orthogonal ubiquitin transfer
p53	Tumor Protein p53
BAX	Bcl-2 associated X protein
PUMA/BBC3	Bcl-2-binding component 3
Rsp5	Reverses Spt- Phenotype 5
FAT4	FAT Atypical Cadherin 4
HEK293T	Human Embryonic Kidney 293 cells
TNF-α	Tumor necrosis factor-α
TRAIL	Tumor necrosis factor ligand superfamily member 10
ERα	Estrogen receptor α
AR	Androgen receptor
SIRT1	NAD-dependent protein deacetylase sirtuin-1
HDAC3	Histone deacetylase 3
NAD	Nicotinamide Adenine Dinucleotide
E1	Ubiquitin-Activating Enzyme
E2	Ubiquitin-Conjugating Enzyme
E3	Ubiquitin Ligases
E4	Ubiquitin Chain Elongation Factor
CO-IP	Co-immunoprecipitation
γ-H2AX	Phospho-Histone H2A.X (Ser139)
CHX	Cycloheximide
Ac-p53	Acetyl-p53
Fas	Fas cell surface death receptor
DR5/TNFRSF10B	TNF receptor superfamily member 10B
p53AIP1	Tumor protein p53 regulated apoptosis inducing protein 1
NOXA/PMAIP1	Phorbol-12-myristate-13-acetate-induced protein 1
qPCR	Quantitative Real-time PCR
HDM2	Human Double Minute 2 homolog
MDM2	Mouse Double Minute 2 homolog
RING	Really Interesting New Gene domain
TPR	Nucleoprotein TPR
Hsp70	Heat shock 70 kDa protein
